# New Names for Three *Penicillium* Strains Based on Updated Barcoding and Phylogenetic Analyses

**DOI:** 10.1128/MRA.00466-21

**Published:** 2021-12-02

**Authors:** Guohua Yin, Wayne M. Jurick, Guozhu Zhao, Joan W. Bennett

**Affiliations:** a Department of Plant Biology, Rutgers, The State University of New Jersey, New Brunswick, New Jersey, USA; b Food Quality Laboratory, USDA Agricultural Research Service, Beltsville, Maryland, USA; c College of Biological Sciences and Biotechnology, Beijing Forestry University, Beijing, China; Vanderbilt University

## REPLY

The genus *Penicillium* contains over 480 species of fungi, and their initial identification is predominantly based on their macroscopic colony morphologies and light microscopic characteristics (1–3). Despite the introduction of modern molecular methods such as the internal transcribed spacer (ITS) sequence and the use of other gene sequences such as β-tubulin (*BenA*) and calmodulin (*CaM*), *Penicillium* species are not easily identified. Mycologists have known for years that there are many inaccurate labels in GenBank for the genus *Penicillium* and other fungal groups (4). In 2020, Houbraken et al. suggested that more diagnostic loci, such as *Cct8*, large subunit (LSU), *RPB1*, *RPB2*, small subunit (SSU), and *Tsr1*, should be included in the classification of *Penicillium* species (1).

In 2015 to 2017, using colony morphology, microscopic characteristics, and the ITS barcode, we identified two *Penicillium* species from stored fruits as Penicillium solitum NJ1 and RS1 (5) and another strain from a flooded home as Penicillium sclerotiorum 113 (6). At that time, we also analyzed the genome sequences of these three culturable strains (7–9). More recently, upon the publication of a letter to the editor on a series of recommendations to the microbiological community to prevent the taxonomic misidentification of genome-sequenced fungal strains suggested by Houbraken et al. (10), we reperformed BLASTn searches using the *Penicillium* strains NJ1, RS1, and 113. We used four barcodes (ITS, *BenA*, *CaM*, and *RPB2*) and limited the query to sequences from type material. With this framework, here we are renaming the three *Penicillium* strains according to the updated BLASTn results.

For *Penicillium* strain NJ1, *BenA* BLASTn results indicated that the top hits were Penicillium crustosum strains NRRL 66388, DI16-101, and CBS115503; among them, CBS115503 is the ex-type strain. The *CaM* BLASTn search for strain NJ1 indicated that the three top hits were P. crustosum strains KrP/6, DTO266-B3, and imi91917 (the ex-type strain). Their maximum/total scores, query coverage, E values, percent identity, and accession numbers are listed in Table 1. Therefore, the *Penicillium* NJ1 strain that we called P. solitum should be renamed P. crustosum NJ1.

**TABLE 1:** Top hits of three misidentified *Penicillium* species using different barcodes

Strain	Barcode	GenBank accession no.	BLASTn hit results						
			Strain description	Maximum score	Total score	Query coverage (%)	E value	Identity (%)	GenBank accession no.
NJ1	ITS	KX243323	**P. crustosum FRR1669** ^*a*^	**1,146**	**1,146**	**100**	**0.0**	**100**	NR077153
			**P. crustosum CBS115503** ^*a*^	**1,108**	**1,108**	**96**	**0.0**	**100**	MH862985
NJ1	*BenA*	KX243332	P. crustosum NRRL 66388	854	854	100	0.0	100.00	KY172962
			P. crustosum DI16-101	854	854	100	0.0	100.00	LT559041
			**P. crustosum CBS 115503** ^*a*^	**837**	**837**	**98**	**0.0**	**99.77**	MN969379
NJ1	*CaM*	KX243340	P. crustosum KrP/6	1,027	1,027	95	0.0	100.00	MW115930
			P. crustosum DTO266-B3	1,005	1,005	93	0.0	100.00	KU711890
			**P. crustosum imi91917** ^*a*^	**1,045**	**1,045**	**99**	**0.0**	**99.26**	DQ911132
RS1	ITS	KX243331	**P. polonicum CBS222.28** ^*a*^	**1,134**	**1,134**	**95**	**0.0**	**99.16**	MH854992
			**P. polonicum NRRL995** ^*a*^	**1,076**	**1,076**	**90**	**0.0**	**99.12**	AF033475
RS1	*BenA*	KX243339	**P. polonicum CBS222.28** ^*a*^	**668**	**668**	**91**	**0.0**	**94.39**	MN969392
RS1	*CaM*	KX243348	P. polonicum F775	979	979	97	0.0	98.84	MG714825
			P. polonicum CMV001E2	977	977	96	0.0	99.03	MK451635
			**P. polonicum CBS222.28** ^*a*^	**946**	**946**	**94**	**0.0**	**98.80**	KU896848
113	ITS	KX365203	**P. maximae NRRL2060** ^*a*^	**1,065**	**1,065**	**88**	**0.0**	**99.28**	NR121343
113	*BenA*	KX365204	P. maximae SFC20151014-M14	795	795	85	0.0	98.59	MK682867
			**P. maximae NRRL2060** ^*a*^	**795**	**795**	**82**	**0.0**	**99.52**	KC773795
113	*CaM*	KX365205	P. maximae SL-CL7	844	844	81	0.0	99.54	MK134677
			**P. maximae NRRL2060** ^*a*^	**829**	**829**	**81**	**0.0**	**99.31**	KC773821
113	*RPB2*	KX365206	P. maximae CBS134565	1,766	1,766	76	0.0	99.67	MN969126

a *Penicillium* type or ex-type strain (in bold).

Similarly, the *Penicillium* strain RS1 *BenA* BLASTn results indicated that the top hit was the Penicillium polonicum ex-type strain CBS222.28, and BLASTn results for the *CaM* sequence of this strain indicated that the three top hits were P. polonicum strains F775, CMV001E2, and CBS222.28 (the ex-type strain). Therefore, this strain should be renamed P. polonicum RS1. Finally, the BLASTn results for the *BenA* and *CaM* sequences of strain 113, which was isolated from a flooded home, indicated that the top hits were Penicillium maximae strains SFC20151014-M14, NRRL2060 (the ex-type strain), and SL-CL7. Therefore, strain 113 should be renamed P. maximae 113. Phylogenetic analyses using genome data from strains NJ1 and RS1 also support new names.

Molecular identification is the default approach for the identification of microfungi (11). With the development of next-generation sequencing, we think that genomics combined with transcriptomics and exometabolomics will provide more diagnostic characters for taxonomically difficult fungi such as *Penicillium* (11, 12).
